# Recent advances in infectious disease research using cryo-electron tomography

**DOI:** 10.3389/fmolb.2023.1296941

**Published:** 2024-01-15

**Authors:** Daniel Asarnow, Vada A. Becker, Daija Bobe, Charlie Dubbledam, Jake D. Johnston, Mykhailo Kopylov, Nathalie R. Lavoie, Qiuye Li, Jacob M. Mattingly, Joshua H. Mendez, Mohammadreza Paraan, Jack Turner, Viraj Upadhye, Richard M. Walsh, Meghna Gupta, Edward T. Eng

**Affiliations:** ^1^ Department of Biochemistry, University of Washington, Seattle, WA, United States; ^2^ Department of Medicinal Chemistry, University of Washington, Seattle, WA, United States; ^3^ Simons Electron Microscopy Center, New York Structural Biology Center, New York, NY, United States; ^4^ Department of Physiology and Cellular Biophysics, Columbia University, New York, NY, United States; ^5^ Department of Molecular Biology and Microbiology, School of Medicine, Tufts University, Boston, MA, United States; ^6^ Department of Physiology and Biophysics, School of Medicine, Case Western Reserve University, Cleveland, OH, United States; ^7^ Department of Chemistry, College of Arts and Sciences, Emory University, Atlanta, GA, United States; ^8^ European Bioinformatics Institute (EMBL-EBI), Cambridge, United Kingdom; ^9^ Department of Microbiology and Immunology, College of Veterinary Medicine, Cornell University, Ithaca, NY, United States; ^10^ Harvard Cryo-Electron Microscopy Center for Structural Biology and Harvard Medical School, Boston, MA, United States; ^11^ Department of Biochemistry and Biophysics, University of California San Francisco, San Francisco, CA, United States

**Keywords:** cryo-ET, pathogen, infectious diseases, viruses, bacteria, host-pathogen interaction, cryo-EM

## Abstract

With the increasing spread of infectious diseases worldwide, there is an urgent need for novel strategies to combat them. Cryogenic sample electron microscopy (cryo-EM) techniques, particularly electron tomography (cryo-ET), have revolutionized the field of infectious disease research by enabling multiscale observation of biological structures in a near-native state. This review highlights the recent advances in infectious disease research using cryo-ET and discusses the potential of this structural biology technique to help discover mechanisms of infection in native environments and guiding in the right direction for future drug discovery.

## 1 Introduction

Electron microscopy is a useful tool to study pathogens, such as intracellular viruses, bacteria, and phages. Historically, transmission electron microscopy (TEM) sample preparation included drying and staining of specimens with a heavy metal. Larger samples, such as tissues or cells, are dried, fixed and embedded in plastic. The fixed block is then sectioned through an ultramicrotome to image via TEM. Although these tools crucial to our understanding of biology, advancements in electron microscopy have eliminated the need for such harsh and elaborate techniques.

**FIGURE 1:**
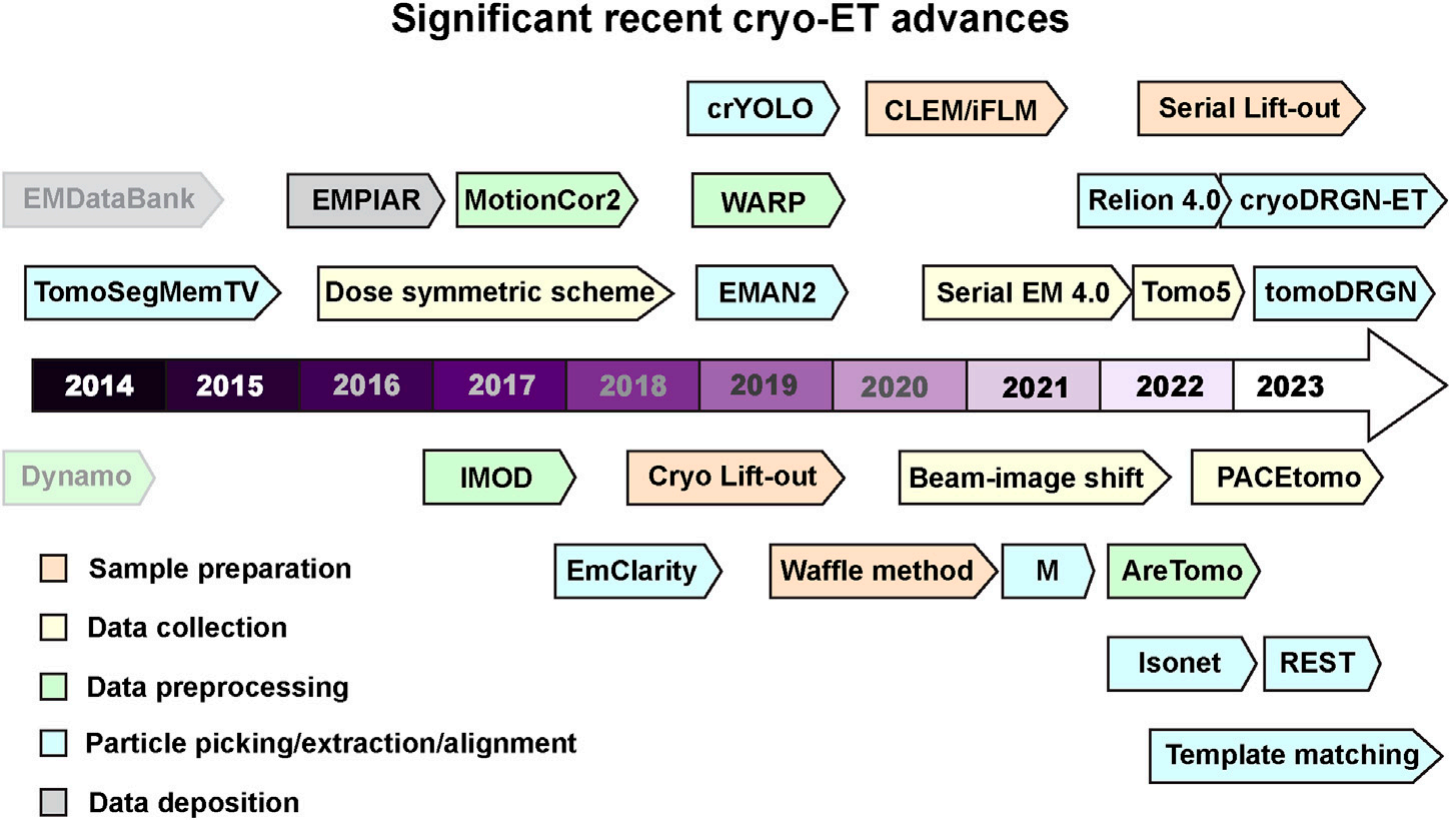
Recent milestones in cryo-ET. Significant advances in tools used for cryo-ET are listed according to the recent timeline. The tools are color coded into various categories- sample preparation, data collection, data processing, and deposition. Dynamo and EMDataBank are greyed out to differentiate that these were developed before the timeline mentioned here. Software packages and tools are outlined to distinguish it from methods and other advances. Several of these tools can perform multiple tasks in addition to their color code provided in the figure. All these cryo-ET developments are discussed and referenced in the main text.

Cryo-EM involves rapid freezing of a sample resulting in it embedded in vitreous ice that is transparent to electrons, and then can be visualized using a TEM. This review covers a sub technique within cryo-EM, cryogenic electron tomography (cryo-ET) that follows many sample preparation steps utilized by traditional cryo-EM, however during data collection, several images are taken of a region of interest at varying stage tilts to generate a tilt-series. After data collection the images can be aligned and reconstructed into a 3-dimensional (3D) volume known as a tomogram. Intracellular pathogens pose an interesting challenge to TEM as electrons can only penetrate 500 nm or less. The advent of focused ion beam (FIB) milling has provided the ability to obtain minimally distorted samples from within prokaryotic and eukaryotic cells by generating thin sections within cells suitable for cryo-ET (Wagner et al., 2020). The rapid advances in cryo-ET are turning it into a technique of choice for cellular structural biology (Figure 1).

**FIGURE 2:**
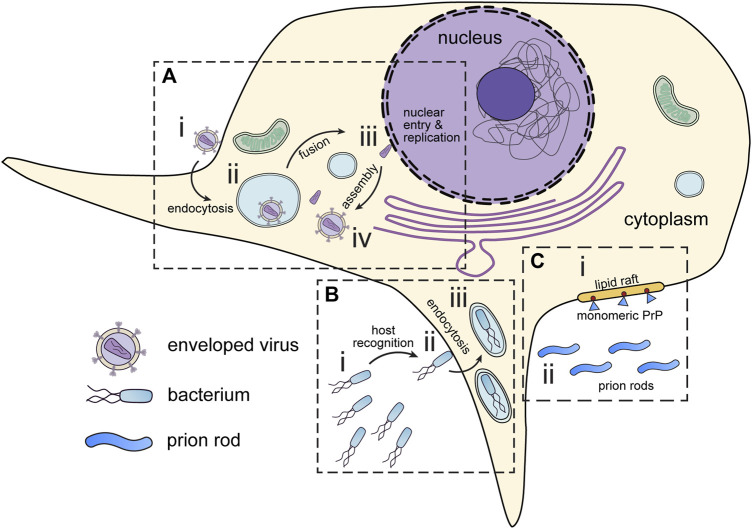
Infectious disease targets for in situ tomography. **(A)** Viruses attach to the periphery of cells (i). Interaction with host organelles, such as the endosomes are sometimes required for entry (ii). DNA viruses and some RNA viruses must transport genetic material to the nucleus (iii) before assembly in the cytoplasm (iv). **(B)** Pathogenic bacteria express motility and virulence machinery which enable them to seek out and infect host cells (i). Upon encountering host cells, bacteria deliver virulence factors which promote host cell entry (ii). Following virulence factor delivery, bacteria enter host cells via endocytosis (iii). **(C)** Prions can interact and seed membranes at lipid rafts on the host cell surface. (i) Monomeric prion proteins (blue) are attached to lipid rafts by glycosylphosphatidylinositol (GPI) anchors (red). (ii) Prion rods occupy the extracellular space. All these have presented interesting targets for *in situ* tomography.

This review will cover the usage of cryo-ET to study pathogens and infectious diseases including but not limited to bacteria, viruses, and parasites (Table 1). As the methods for cryo-ET sample preparation, data collection, and processing continue to improve, researchers now have the potential to achieve high-resolution imaging of both bacterial and eukaryotic parasites within host cells (Figure 2). One approach is the direct observation of host-pathogen interactions at thin cell edges (<300 nm), which does not require complex sample preparation. Host-pathogen interaction at different time points can we visualized, gaining valuable insights into the mechanisms of infection and disease progression (Chmielewski et al., 2022). However, much of the life cycle of parasitic pathogens takes place within the thicker host cell interior. In such cases, the samples can be too thick to obtain high-quality tomographic data without sample thinning using Cryo-Electron Microscopy of VItreous Sections (CEMOVIS) or cryo-FIB milling (Al-Amoudi et al., 2004; Marko et al., 2007). This opens new avenues for studying the structural details of host-pathogen interactions, the spatial and temporal organization of intracellular pathogens, and the dynamic processes occurring during infection and pathogen life cycle.

**TABLE 1:** A summary of recent publications utilizing cryo-ET to study viruses, bacteria, and prions. FIB: focused ion beam; TOMO, tomography; SPA, single particle analysis; Subtomogram averaging, STA.

Pathogen	Stage/activity	Structure	Methods	Citation
*Rotavirus*	Assembly Intermediates	VP4	SPA/FIB/TOMO/STA	Shah et al. (2023)
*Chikungunya virus*	Budding	Capsid	TOMO/STA	Chmielewski et al. (2022)
*Chikungunya virus*	Fusion/Entry	E1	SPA/TOMO/STA	Mangala Prasad et al. (2022)
*Human immunodeficiency virus (HIV-1)*	Nuclear Transport of capsid	Capsid/Nuclear pores	FIB/TOMO/STA	Schur et al. (2016), Zila et al. (2021)
*Toxoplasma gondii*	Host invasion	Tubulin	TOMO/STA	Sun et al. (2022)
*Plasmodium berghei*	Motion	Cytoskeleton	TOMO	Kudryashev et al. (2010)
*Plasmodium falciparum* and *Plasmodium berghei*	Biosynthesis	Apicoplast	TOMO	Lemgruber et al. (2013)
*Theileria annulata*	Pathogen–host interaction	Filamentous membrane protrusion	TOMO	Kühni-Boghenbor et al. (2012)
*Cryptosporidium parvum* and *Toxoplasma gondii*	Invasion and infection	Rhoptry	TOMO/STA	Mageswaran et al. (2021)
*Trypanosoma brucei*	Motion	PFR, PFR-axoneme connectors, Axonemal complex	TOMO/STA	Zhang et al. (2021)
*Chlamydia trachomatis*	injects virulence effector proteins into host cells	Type III secretion system	TOMO/STA	Nans et al. (2014)
*Coxiella burnetii*	inject effector proteins into host cells	Type IV Secretion System	FIB/TOMO/STA	Park et al. (2022)
*Salmonella enterica*	Host immune response	Guanylate-binding protein	FIB/TOMO/STA	Zhu et al. (2021)
*263k prion*	Replication	Prion rods	SPA	Kraus et al. (2021)
*ME7 prion*	Replication	Prion rods	SPA	Manka et al. (2023a)

## 2 Achievements and limitations of cryo-ET to explore infectious diseases

### 2.1 Study of the viral replication cycle with cryo-ET

All viruses contain a genome protected by a biological barrier. These two simple components can be any variety of biological molecules with viruses utilizing RNA and/or DNA and proteins. This review discusses prions as infectious agents (Section 1.3). While acknowledging the historical view, we do not consider prions as viruses in this regard (Prusiner, 1998). Viral genomes can be single or double stranded nucleic acids, and their biological barrier can range from a single membrane or protein layer to several proteinaceous cores and/or multiple membranes (Flint et al., 2020). Most viruses range from ∼20–1,000 nm making analysis via standard light microscopy impossible. Early structural studies were carried out on viral samples that have been purified from the host, as reviewed in (Roingeard, 2008). These early samples consisted of very high titer virus produced in eggs or from human samples such as feces or infected tissue but had very high background protein in addition to the virus of interest. Although the resolution was poor, these studies were the first glimpses into the molecular architecture of viruses. In the decades following, electron microscopy became a useful diagnostic tool and has been used for identification and classification of new or emerging viruses. For example, identification of the first Severe Acute Respiratory Syndrome (SARS) virus that emerged in Hong Kong and Southern China in 2003 as a coronavirus was due to TEM (Ksiazek et al., 2003). Since 1990s, TEM of purified virus as a means for identification has been supplanted by higher throughput nucleic acid detection strategies such as ELISA or PCR (Roingeard et al., 2019). Although diagnostic applications have diminished, purified viruses are still commonly used in cryo-EM studies to generate high resolution structures.

Fewer studies exist of viruses within their host cell. In the 1950s, ultramicrotomes were first used to generate thin sections of cells that could be imaged with conventional TEM (Winey et al., 2014). However, ultramicrotomy requires dehydration, staining, and plastic embedding, to enhance contrast, but can extensively remodel membranes and other cellular features. It should be noted that this approach still provides most preliminary overviews or phenotypes in virology, and cryo-EM has not yet been broadly employed for non-structural phenotypic analysis.

Studying viral infections *in situ* using cryo-ET has allowed researchers to gain new insights into the machinery of pathogens and cell biology of infection. Viruses manipulate host membranes at many steps of their replication cycle: entry, assembly, and egress-

#### 2.1.1 Viral entry

Viral entry is facilitated by factors on the surface of the virus interacting with factors on the host cell surface. Non-enveloped virus entry is often achieved through the hijacking of cellular entry mechanisms such as clathrin-mediated endocytosis (Anderson et al., 1996) as utilized by SV40, or caveolar endocytosis at tight-junctions (Coyne et al., 2007) in some adenoviruses. Enveloped virus entry can also occur through cell mediated endocytosis and/or direct membrane fusion. For example, Ebola virus entry is facilitated first by macropinocytosis of the entire virion into an early endosome which the virus then escapes from, using its type 1 fusion protein GP1 (Salata et al., 2019). Regardless of the virus, the viral entry process can be complex, and comprises several steps.

Structural studies regarding the proteins involved in viral entry are of incredibly high interest for basic virology as well as vaccinology. Non-enveloped as well as enveloped viral entry proteins are always found on the surface of the virus and these proteins tend to be highly immunogenic and can serve as promising vaccine candidates (Narkhede et al., 2021). The type 1 fusion proteins utilized by viruses such as HIV, SARS-CoV-2 and Influenza have all been visualized at high resolution through crystallography and cryo-EM single particle analysis (SPA). However, due to technical limitations, a vast majority of structures have been substantially altered either by mutagenesis or truncations to stabilize the proteins of interest (Rey and Lok, 2018). Nonetheless, these structures have been pivotal in the development of monoclonal antibody therapeutics as well as vaccines. However, information regarding the physiologically relevant conformations and function of these proteins is lost. Cryo-ET studies, either *in vitro,* or *in situ*, would allow for the functional characterization of viral entry proteins in their native, unedited state. Many enveloped viruses require one or more factors for fusion to occur, the Influenza virus entry protein haemagglutinin (HA) can be triggered by low pH alone (Sieczkarski and Whittaker, 2005). *In vitro* cryo-ET studies have captured key hemifusion intermediates by mixing virus or virus-like particles containing HA with liposomes at a low pH, thus, allowing the analysis of functional intermediates at high resolution, commonly lost in traditional SPA analysis (Calder and Rosenthal, 2016; Chlanda et al., 2016; Gui et al., 2016). These studies shed light on the elusive entry strategies utilized by enveloped viruses. Similar studies on non-enveloped viruses using cryo-ET are fewer but have made a significant impact on the understanding of their entry mechanism. A membrane perforation in the host cell is required for a non-enveloped virus to enter mediated by the virus outer-layer protein. A combination of SPA and cryo-ET experiments on rhesus rotavirus (RRV) determined that a complete conformation change of the outer-layer protein (VP4) from “upright” to a “reversed” is necessary for viral entry (Herrmann et al., 2021). Another report on a non-enveloped virus Bluetongue virus (BTV) entry shows that the membrane penetration protein (VP5) changes conformation upon interaction with the liposomal membrane that primes the virus for membrane penetration. The shortening of VP5 stalk length signifies the viral entry into the membrane as shown by cryo-ET. The authors also show this stalk interaction at low pH results in single pores in the membrane (Xia et al., 2021).

#### 2.1.2 Viral assembly

Perhaps the most applicable use case of *in situ* cryo-ET in virology is to study viral assembly and budding. Inhibiting the viral fusion/entry machinery has historically been the most popular mechanism for vaccine and therapeutic design, however, due to the high amount of evolutionary selection these proteins undergo they may not always be the best target. The next step of the viral life cycle that can be targeted is viral assembly and egress. This has been most recently demonstrated by Lenacapivir, a drug that binds the HIV-1 capsid directly and stabilizes the lattice has recently been FDA approved (Bester et al., 2020; Segal-Maurer et al., 2022). Development of such drugs would not be possible without the decades of structural studies that preceded, understanding the capsid lattice of HIV (Graham and Zhang, 2023). Both *in vitro* as well as *in situ* cryo-ET methods have been pioneered through the study of the HIV capsid lattice (Schur et al., 2016; Zila et al., 2021). Historically, HIV-1 is one of the most well-funded and studied viruses of all time, however, the COVID-19 pandemic led to increased focus on SARS-CoV-2. Within a year of the pandemic, studies of SARS-CoV-2 viral assembly and budding from *in situ* cryo-ET were published and quickly transitioned into other viruses (Klein et al., 2020). Recently, Ebola virus in-cell nucleocapsid assembly mechanism was determined using cryo-ET. Polymerized nucleoprotein (NP) condenses on the RNA genome with other viral proteins protecting the viral genome making the core of filamentous Ebola virions (Watanabe et al., 2023).

The structures generated in these studies highlight the true power and potential of *in situ* cryo-ET, generation of these samples in an *in vitro* biochemically pure context would be incredibly difficult if not impossible.

### 2.2 Bacterial and parasitic diseases

Cryo-ET also has promise as a method for better understanding bacterial and parasitic diseases. To combat these diseases, it is important to understand how these pathogens move through their environment to seek out host cells, interact with host cells, deliver virulence factors to their hosts, and grow and divide within them.

#### 2.2.1 Pathogen motility systems and cytoskeleton

To grow and reproduce, intracellular bacterial and eukaryotic pathogens, which are often obligate parasites, must navigate their environments to encounter suitable host cells. This often entails navigation through turbulent or viscous media like blood, and these pathogens must use specialized organelles like flagella to move through them. Because of the importance of cell motility for the ability of intracellular pathogens to infect their hosts, much attention has been given to determining the structures of motility systems and cytoskeletal features. Due to its ability to image large cellular features, cryo-ET has served as a useful tool in characterizing the structures of parasite flagella and related organelles. For example, a 2021 study used cryo-ET to examine the structure of the *Trypanosoma brucei* paraflagellar rod (PFR) as well as the connections between the PFR and axoneme, the core structure of the flagellum (Zhang et al., 2021). The PFR is an essential feature of the *Trypanosoma* flagellum which is crucial for non-planar motion of this parasite in its medium as well as for signaling involved in host-pathogen interactions. Cryo-ET with subtomogram averaging (STA) enabled the determination of the repeating, scissor-like structure of the PFR as well as the structures of the accessory filaments connecting the PFR to the axoneme. Similar methods have since been used to determine the structures and identities of microtubule inner proteins involved in *T. brucei* motility (Shimogawa et al., 2023).

Cryo-ET has enabled the characterization of pathogen cytoskeletal arrangements, which are critical both for cell motility and for interactions with the host. Subnanometer structures of tubulin-based components of the parasite *Toxoplasma gondii* are recently determined (Sun et al., 2022). *T. gondii*, the causative agent of toxoplasmosis, is a prevalent intracellular pathogen which must maintain cellular shape to move through its environment and organize machinery for host cell invasion, both of which are accomplished using tubulin-based cellular fibers. To determine structures of these fibers, Sun et al., 2022, imaged both detergent-extracted *Toxoplasma* cells and thin edges at the conical cell anterior, which houses the machinery responsible for interacting with the host cell during invasion. Using STA, the researchers were able to obtain subnanometer reconstructions, allowing them to identify the geometry of these tubulin fibers as well as locating the individual tubulin subunits which comprise them.

#### 2.2.2 Pathogen secretion systems and host Cell invasion

Many bacteria and eukaryotic microbes are obligate parasites which carry out their life cycles in distinct stages. Host cell entry is initiated by the delivery of virulence factors to the host cell by pathogen membrane-associated nanomachines called secretion systems. These systems are large, multiprotein complexes, and the structures of some of the components of these systems have been determined using SPA on purified samples. The ability to determine structures of secretion systems interacting with host membranes enabled by cryo-ET is an exciting new development.

Recently, cryo-ET was used to determine structures of secretion systems in eukaryotic parasitic microbes from the phylum Apicomplexa, which cause diseases such as toxoplasmosis and malaria (Lemgruber et al., 2013; Mageswaran et al., 2021; Martinez et al., 2022; Gui et al., 2023; Martinez et al., 2023). These parasites use cellular organelles called rhoptries to deliver virulence factors to host cells and initiate invasion. Because of the crucial role of the rhoptries in the process of infection, better understanding their structure, function, and organization within parasite cells is important. Structures of various Apicomplexan rhoptries have been determined via cryo-ET, including those from the parasites *Toxoplasma gondii*, *Cryptosporidium parvum*, and *Plasmodium falciparum*, permitting the identification of species-specific differences in rhoptry organization. Additionally, the use of cryo-ET data processing strategies including subtomogram extraction, classification, and averaging have permitted the identification of various rhoptry states prior to infection, revealing additional information about how rhoptry-associated cellular structures e.g., apical vesicle, may participate in priming the rhoptries for virulence factor delivery prior to parasites encountering host cells.

Following virulence factor delivery to host cells by secretion systems, intracellular parasites and bacteria undergo host cell invasion via endocytosis. This involves interactions between host and pathogen membrane-embedded factors and a remodeling of the host cell membrane and cytoskeleton. Because host-pathogen interactions during infection take place at cell edges, where cells are most likely to be thin enough to transmit an electron beam when cultured on TEM grids, it is possible to use cryo-ET to capture these interactions alongside their associated host cytoskeletal rearrangements, giving a clear picture of the processes involved in pathogen endocytosis. A 2014 study of *Chlamydia* type-III secretion systems (T3SS) determined both the structure and arrangement of T3SS machinery on infectious *Chlamydia* elementary bodies as well as the interaction of elementary bodies with host cells, capturing the arrangement of host cell actin filaments at the host-pathogen interface during the process of endocytosis (Nans et al., 2014). Cryo-ET serves as an increasingly important tool for characterizing the process of infection by many bacterial and eukaryotic pathogens.

### 2.3 Prion diseases

Prion diseases, or transmissible spongiform encephalopathies (TSEs), are the only infectious neurodegenerative disease (Prusiner, 1998). These pathogens consist of a single protein (Prusiner, 1982), prion protein (PrP), which is ubiquitously expressed in the brain of normal uninfected animals (Chesebro et al., 1985). The infectious agent (prions), or the scrapie form of PrP (PrP^Sc^), however, is chemically equivalent to the normal cellular PrP (PrP^C^) but differs in conformation. Further investigation has revealed that prions are amyloid fibrils (Nguyen et al., 1995), and their propagation can be explained by the nucleation-dependent polymerization model, in which the pathogenic PrP^Sc^ converts the normal PrP^C^ into PrP^Sc^ and thus elongates (Jarrett and Lansbury, 1993). The defining feature of amyloids is their cross-β structure, which arises from the arrangement of β-strands in an orientation perpendicular to the axis of the fibril, with an interval of ∼4.8 Å (Eanes and Glenner, 1968).

#### 2.3.1 Structural biology of PrP

The spontaneous generation and propagation of prions is suitable for structural biology studies as it involves only conformational changes. Currently, various methods have been used to determine both PrP^C^ (Riek et al., 1996) and some strains of PrP^Sc^ (Artikis et al., 2022; Caughey et al., 2022; Manka et al., 2023b).

PrP^C^ is a highly conserved, small glycoprotein tethered to the extracellular surface of the plasma membrane by a C-terminal Glycosylphosphatidylinositol (GPI) anchor (Stahl et al., 1987; Haraguchi et al., 1989). Based on solution NMR data, monomeric PrP can be divided into two parts, a disordered N-terminal region and a folded C-terminal part consisting of two β-strands and three α-helices with a disulfide bond linking α-helices 2 and 3 (Riek et al., 1996).

However, our understanding of the structure of PrP^Sc^ is still limited, with the first high-resolution structure of brain-derived infectious mammalian prion only recently reported in 2021 by cryo-EM (Kraus et al., 2021). The reported prion structure adopted the same parallel in-register β-sheet structural architecture like most other non-infectious amyloid fibrils of PrP (Glynn et al., 2020; Wang et al., 2020; Wang et al., 2021; Li et al., 2022a; Chen et al., 2022), thus it will take further investigation to understand what structural features make prions infectious. Subsequently reported high-resolution structures of other mammalian prions showed a similar three β-arch structure (Hoyt et al., 2022a; Hoyt et al., 2022b; Manka et al., 2022; Manka et al., 2023a), in agreement with the fact that they are all propagated from the same strain using different lab animals. The progress of such studies are largely limited by multiple challenges in sample preparation and data processing (Zielinski et al., 2021).

#### 2.3.2 Cryo-ET studies of prions

The high-resolution structures of prions have provided valuable insights into this pathogen and have the potential to aid in development of diagnosis and therapeutic strategies. However, despite these advances, the most fundamental aspect of prion infection, the mechanism by which prions cause neurodegeneration, remains poorly understood. The current *ex vivo* structures do not fully explain the complex processes including transportation within the circulation system, penetration of the blood-brain barrier, and causing spongiform degeneration. Thus, cell-based systems are essential, even though prions are capable of propagating without the assistance of a functional host cell (Castilla et al., 2005).

So far, cryo-ET has only been used to determine the helical handedness of *ex vivo* prion rods (Kraus et al., 2021; Hoyt et al., 2022a; Hoyt et al., 2022b). Since the propagation of prions involves GPI modified PrP^Sc^ and PrP^C^, and the latter is tethered to the lipid rafts, it is critical to focus on the membrane surface to study the propagation process. Due to the helical nature of PrP^Sc^ and hence the helical arrangement of GPI molecules, it has been speculated that membranes tend to wrap around PrP^Sc^ (Kraus et al., 2021), or even form a tube. Such processes would be a suitable target for cryo-ET studies. Compared to other pathogens of infectious diseases, infectious prions must be prepared from infected animals with limited yield and purity, making it a major challenge for cryo-ET studies.

### 2.4 Cryo-ET is a tool to better understand the infection mechanism

Infectious diseases are instigated by pathogens that infiltrate the body, reproduce, and trigger illnesses. This intricate process involves the intertwining biochemical pathways of both the pathogen and the host. *In-situ* approach emerges as a more practical option for such investigations. Particularly, for less extensively studied pathogens lacking specific molecules or targets, observing pathogens within host cells can offer initial insights into the infection process. The integration of STA could potentially unveil crucial target molecules or even identify them (Winter and Chlanda, 2021). Even for well-studied systems where intricate processes can be simplified as *in vitro* reactions, *in situ* cryo-ET presents a more natural environment.

In summary, the utilization of *in situ* cryo-ET yields a potent investigative tool for uncovering the structural underpinnings of infectious diseases. It empowers researchers to explore live pathogens within their host cells, capturing essential molecular interactions and gaining valuable insights into the infection mechanism. Moreover, the fusion of cryo-ET with STA techniques holds the promise of high-resolution structural analyses, paving the way for the advancement of targeted interventions and therapeutic strategies.

## 3 Sample preparation and hardware

### 3.1 Sample preparation: plunge freezing without requirement for thinning

**FIGURE 3:**
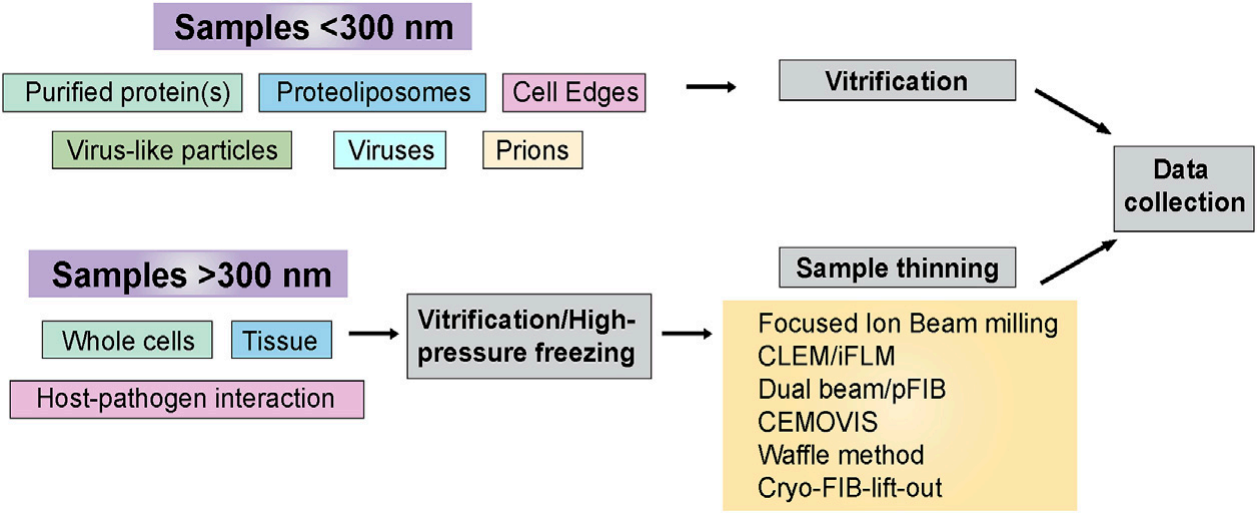
Sample preparation flowchart. Sample thickness is an important determinant in deciding the strategy for cryo-ET sample preparation before data collection. This flowchart outlines the freezing methods and post-freezing sample prep requirements before data acquisition.

At times, cryo-ET sample preparation can be accomplished using straightforward blotting and plunge freezing techniques. This is particularly true when the samples of interest are thin, such as purified protein(s), VLPs, cell edges, and prions. Many crucial steps in pathogen life cycles occur within the interiors of host cells, which may be too thick to collect high-quality cryo-ET data. Some of the approaches are mentioned later in the sample thinning Section 2.2. Figure 3 outlines sample types and sample preparation approaches based on their complexity.

#### 3.1.1 Purified proteins

Cryo-ET may also be useful for purified samples such as protein complexes reconstituted *in vitro*. Tomograms potentially represent an entire sample volume, including the top and bottom surfaces, and enable precise determination of sample thickness, residual tilt, and particle distribution within the sample. These data are especially useful in sample preparation methods development, and cryo-ET has been used to quantify particle adsorption to air-water interfaces during plunge freezing (Noble et al., 2018) as well as novel substrate materials such as monolayer graphene oxide (Palovcak et al., 2018). Cryo-ET also provides an empirical approach to obtain initial models for SPA, much as random conical tilt was used before the advent of current-generation *ab initio* reconstruction algorithms for projection images based on stochastic gradient descent. One such example where cryo-ET helped get better mechanistic information from a purified protein complex is RNA polymerase II preinitiation complex (PIC) and Mediator assembly on a divergent promoter (Gorbea Colón et al., 2023). The authors were able to visualize DNA organization with the proteins using cryo-ET that was otherwise not clear using SPA.

#### 3.1.2 Virus-like particles (VLPs)

VLPs are an effective and safe way of directly observing the different stages of viral infection while retaining the context of the whole virus. For example, HIV VLPs can be produced by transfecting cell lines that stably express its glycoprotein receptor responsible for driving attachment and fusion: Envelope glycoprotein (Env), with an Env-deficient HIV-1 backbone plasmid, which contains all the additional components that virions require to assemble and bud. Env is incorporated onto VLPs during budding, but because the plasmid does not contain the Env gene, these VLPs are replication incompetent. By mixing proteoliposomes with VLPs under controlled *in vitro* conditions, numerous studies have captured intermediate fusion states over a time course and characterized multiple viral fusion pathways (Schur et al., 2016).

SARS-CoV-2 VLP protocols are well optimized and being used to assess the infection steps (Syed et al., 2021). VLPs with a specific Spike variant have been used to test binding and assess epitopes of two human antibodies from the blood of a convalescent donor using cryoET (Li et al., 2022b). Target extracellular vesicles containing human hACE2 with suitable tags has been used with SARS-CoV-2 VLPs to study viral fusion. The authors were able to map intermediate Spike stages and a late fusion conformation using cryo-ET (Marcink et al., 2022).

#### 3.1.3 Purified viruses

Although VLPs provide an excellent model system for the study of individual viral components, oftentimes intact virus particles offer a more physiologically relevant system. Once a virus infects a cell, to be transmissible and survive it must successfully exit the host cell to infect a new cell. Viruses do this either through the cell lysis, inducing budding through cellular membranes and/or hijacking exocytosis pathways. This viral trait can prove useful or be an obstacle when it comes to purification for electron microscopy. Some enveloped viruses can bud out of cells while keeping the host cell intact. For these viruses, purification can be as simple as a single round of ultracentrifugation where the supernatant from infected cells can be quickly cleared, and virus can be concentrated (Gias et al., 2008). However, most non enveloped viruses and many enveloped viruses can lead to severe cytopathic effects and subsequent lysis of cells. In these cases, there is a requirement for more elaborate purification methods prior to electron microscopy. Nonenveloped viruses tend to be more stable and can survive the methods commonly associated with protein purification such as lysis, detergents, and column purifications (Huhti et al., 2013; Florea et al., 2023). This is rarely the case with enveloped viruses as they can be quite fragile, and purification might perturb physiologically relevant conformations. Some groups have tried to reduce damage to enveloped viruses introduced by ultracentrifugation by adopting an on-grid binding approach (Kiss et al., 2014). Utilizing an affinity tagged virus (6xHis or 3x Flag), grids were coated in the appropriate capture molecule (Ni-NTA or anti-Flag IgG), the authors were able to capture and image members of the *Retroviridae*, *Orthomyxoviridae*, and *Paramyxoviridae* families. Traditional plunge freezing can be used for cryo-EM vitrification as the diameter of most viruses is small enough for ice thickness to not become an issue.

#### 3.1.4 Cell-edges

As an early example of cryo-ET of thin cell-edges, Nans, et al., used cryo-ET in a 2014 study to observe interactions between *Chlamydia trachomatis* elementary bodies and cultured human cells. *Chlamydia trachomatis* is an obligate intracellular parasite which causes sexually transmitted infections in humans, and it must invade host cells during the infectious “elementary body” stage of its life cycle to grow and reproduce. The edges of adherent human cells can be sufficiently thin to transmit an electron beam, which enabled researchers to observe interactions between the *Chlamydia* type-III secretion system and host cells at the early stages of infection, as the elementary bodies are endocytosed (Nans et al., 2014).

Attempts to look at viral infection events at host cell edges have been successfully made. Chikungunya virus assembly observed at the host cell edges using cryo-ET revealed 12 intermediate states (Chmielewski et al., 2022). CHIKV vaccine strain 181/clone 25 (CHIKV-181) was used to infect U2OS cells grown on fibronectin coated gold 200 mesh grids. Grids were frozen with 10 nm BSA gold tracer. A Volta phase plate was used with the TEM to enhance the contrast in the images. In a similar way, *in-situ* visualization of SARS-CoV-2 vaccine ChAdOx1 nCoV-19 products on host cell edges revealed the receptor-binding domain (RBD) status of the spike proteins (Ni et al., 2023). In both cases, STA and classification played an important role in improving resolution and identifying macromolecular heterogeneity.

#### 3.1.5 Prions

The optimal system for studying prions is adherent primary neuronal cell cultures, given that prions’ native host cells are neurons (Cronier et al., 2004). Furthermore, the width of these axons and dendrites allows for direct electron imaging (Foster et al., 2021), minimizing thinning concerns. Nonetheless, infecting cells with prions, which requires animal or patient-derived prions, and prolonged incubation periods is a significant challenge (Cronier et al., 2004). Notably, fluorescence labeling of PrP often disrupts the seeding reaction, complicating the real-time observation of infection events. These factors contribute to the delayed progress *in situ* prion infection studies compared to research on other pathogens. However, in contrast to many other pathogens, amyloid fibrils including prions exhibit remarkable resilience. These protein filaments can be extracted *ex vivo* using detergents and harsh conditions, undergoing extensive sample preparation while retaining high-resolution structural integrity (Fitzpatrick et al., 2017; Kraus et al., 2021; Manka et al., 2022). Recent cryo-ET investigations of *in situ* amyloid fibrils from high-pressure frozen tissue (Gilbert et al., 2023) have demonstrated near-identical structures to those observed *ex vivo* (Fitzpatrick et al., 2017). Thus, for structural studies involving prions or other amyloid fibrils, it is more practical to use *ex vivo* samples for high-resolution SPA studies while *in situ* cryo-ET studies should focus more on exploring their biological interactions.

### 3.2 Sample preparation for thicker samples: thinning methods

#### 3.2.1 On-grid thinning

Bacterial and fungal pathogen cells can be deposited directly on grids after growth, or any treatment required in suspension cultures. In one example, 4 µL of the bacteria suspension culture was applied on glow-discharged, thick carbon-coated, 200 mesh grids. Excess fluid was blotted away in a 100% humidity chamber and the grid was plunge-frozen in a mixture of ethane/propane (Kaplan et al., 2021). Cryo-ET on bacterial samples is reviewed here documenting variations in the sample preparation (Khanna and Villa, 2022).

Host adherent mammalian cells can be grown on cryo-EM grids treated with various substrates like fibronectin, poly-Lysine, and collagen that form an extracellular matrix (ECM) to support attachment of cells (Wagner et al., 2020; Franken et al., 2022). Copper cryo-EM grids are toxic to the cells hence other materials like gold, silicon oxide, are preferred (Han et al., 2012). Note that C-clip and rings for autoloaders are copper and pre-clipped grids are thus unsuitable for cell culture unless these materials are gold plated in-house or by manufacturers on special request. It is desirable to have cells distributed on the grids evenly e.g., one to two cells per square away from the grid bars. Clusters of mammalian cells can lead to non-vitreous ice. Cell strainers of various pore sizes can be useful to achieve singly dispersed cells. Micropatterning on cryo-EM grids with the ECM substrates can also be used to have targeted attachment of the cells on the grids (Sibert et al., 2021a; Sibert et al., 2021b).

• Sterile technique. Culturing cells on EM grids necessitates unusual operations such as touching of culture surfaces with forceps. “Best effort” sterile technique should be used including ethanol treatment of forceps and soft UV treatment of other materials (e.g., leaving under a biosafety cabinet UV light for 15–60 min). Glow discharge or plasma cleaning seems to provide sufficient sterilization of grids, but ethanol treatment may be required for the slide or block and dishes used for grid transport. Finally, it may be helpful to add antibiotics to culture media at least during grid preparation and cell seeding.
• Grid types
   ○ 200 mesh grids are suitable for eukaryotic cells, as 300 and finer mesh grid squares prohibit cell spreading. Finer mesh can be problematic as the grids bars can be in the way of sample thinning and imaging.
   ○ Ideal grids for cell periphery imaging (without thinning) allow good adhesion with a significant imageable area (holes). Recommended types: holey carbon with 3.5/1 µm spacing and thin carbon layer, holey carbon with 2/2 or 2/1 µm spacing with or without thin carbon layer.
   ○ Ideal grids for cryo-FIB-SEM applications allow good cell adhesion and robustness, with sufficient space for a shallow angle ion beam to pass the grid bars. Grids should be 200 mesh, continuous carbon on formvar, or any holey carbon surface (spacings are unimportant as any substrate below milled lamellae is destroyed).
   ○ Purpose made grids are required for lift-out applications, usually in the form of “half-moon” grids featuring a series of posts for affixing lamellae.
• Choice of ECM substrate
   ○ poly-D-lysine, collagen, and fibronectin are all suitable. Fibronectin is probably most used currently. Typically, fibronectin is diluted to 1–25 μg/mL in cell culture water or PBD, and grids are coated analogously to a preparation for negative stain EM. Droplets of ∼40 µL are placed on parafilm, and glow-discharged grids are floated face-down (1 per droplet) for 15–30 min before being blotted with filter paper, rinsed once with diluent, and blotted as dry as possible before being placed face-up in a culture dish for cell plating. Higher concentrations of ECM substrate deliver stronger cell adhesion, while lower concentrations favor spreading and thinning of cell peripheries (author observation).
• Adding cells to grids
   ○ Optimal culture protocols will be unique to each cell type, but the following guidelines based on U2OS cells provide a starting point for mammalian cell lines.
   ○ Direct seeding: a dense suspension of cells is applied directly to grids, for example 5 μL at ∼500 cells/µL and are allowed to adhere for 5–30 min before careful addition of culture media.
   ○ Dilution and plating: a dilute suspension of cells is carefully added to an appropriate volume and total cell count. For example, if grids are placed in 6-well dishes (9.6 cm^2/well), approximately 30,000–100,000 cells in 2 mL of media should be added to each well (author observation).
• Vitrification
   ○ Cells are sensitive to front-side blotting, which leads to unique pressure-derived phenotypes, and can potentially remove adhered cells.
   ○ Back-side blotting can be performed most easily using a manual plunger or a Leica GP2 automatic plunger. A Vitrobot may be used either manually using a strip of folded filter paper held by locking forceps, or after modification by removal of one blotting arm.
   ○ Rinsing using isosmotic buffer (e.g., PBS for cell culture) can be helpful in reducing background from supplemented (serum-containing) media.
   ○ Grids withdrawn from culture media often carry only small amounts of liquid and an additional 0.5–3.5 µL of liquid may be helpful in achieving contact with filter paper and driving blotting. Small volumes added to the back of the grid are especially useful for back-side blotting.
   ○ Near-complete removal of liquid is usually desirable, and back-side blotting is less efficient than front-side or two-sided blotting, thus long blotting times of 10–15 s are common (author observation).

#### 3.2.2 CLEM/iFLM

Cryo-ET provides the means to determine *in situ* structural information at high resolution, but identifying regions of interest at low magnification can be exceptionally challenging. This challenge increases dramatically when the complex of interest is transient or simply low in abundance or both. Correlative light and electron microscopy (CLEM) aims to pair localization derived from fluorescence tagged regions of interest, with electron microscopy. While the idea here is rather simple: find and map a fluorescence signal(s) in a cell on a cryo-EM grid and then collect tomographic data at this location(s); there are several logistical challenges that had to be overcome and are continuing to improve. The cells must be deposited or grown on a grid, successfully frozen in vitreous ice, kept at cryogenic temperatures in a dry environment while being shuttled from the various instrumentation required to go from an identified fluorescence signal to a tilt-series collected on a thin lamella in a TEM. Adaptations to incorporate cryo-stages in light microscopy instruments (and changes in optics), shuttling devices to keep samples dry and at cryogenic conditions, and software that permits correlation of identified regions of interest to flow to each step in the workflow were paramount in making this procedure possible. An additional adaptation, integrated fluorescence light microscope (iFLM), places a fluorescence microscope within the chamber of the cryo Focused Ion Beam Scanning Electron microscope (FIB-SEM) reducing the need for one of the transfer steps. The result of these software and hardware integrations is an identified region of interests from FLM on a frozen hydrated sample that is then milled in a focused Ion beam SEM instrument and then transferred to a TEM to collect high resolution information, while maintaining temperature and a dry environment through each step of the process. Improvements that increase the fidelity of maintaining ideal sample conditions and ease of each step of the process are continual areas of innovation (Fu et al., 2019; Gorelick et al., 2019; Yang et al., 2023a).

There is a resolution gap between traditional cryo-fluorescence light microscopy (cryo-FLM) and cryo-EM, ∼400 nm limitation in cryo-FLM compared to sub nanometer information for cryo-EM. Cryo super-resolution light microscopy aims to reduce that gap, producing localization information in the neighborhood of 30 nm (Tuijtel et al., 2019; Dahlberg and Moerner, 2021).

#### 3.2.3 CEMOVIS

An important historical development for the imaging of biological specimens with electron microscopes was the development of vitreous sectioning, permitting the visualization of thinned cell or tissue samples in a hydrated state and in the absence of heavy metal stains. Cryo-electron microscopy of vitreous sections (CEMOVIS) involves first high-pressure freezing thick biological samples to form vitreous ice, followed by sectioning with a cryo-microtome to render samples thin enough to transmit an electron beam. Thinned sample sections can then be applied to TEM grids with pressure to prepare them for imaging. Sectioned samples can range in thickness from the low hundreds of nanometers to as little as 50 nm, allowing the collection of tilt series data for tomography. While it allows for the collection of TEM data on hydrated, unstained samples, CEMOVIS has drawbacks related to the use of a microtome for sample thinning. The sectioning process applies physical stress to the sample, which can lead to the formation of compression artifacts, distorting the shape and size of sample features. Additionally, the microtome knife can damage the sample through the formation of crevasses during thinning. In cases where physical deformation of the sample cannot be tolerated or the sample of interest is too small for cryo-sectioning, other thinning techniques such as focused ion beam milling may be preferred (Al-Amoudi et al., 2004).

#### 3.2.4 Dual beam/pFIB

Cryo-ET requires specimens that are thin enough to be imaged in a TEM. This places an upper limit on sample thickness in the range of 300–400 nm (Lučić et al., 2013). To utilize *in situ* cryo-ET one must image the thin edge of an intact cell or thin the sample to an acceptable thickness for TEM imaging. This thinning of cells into a useable lamella has mostly been accomplished with a gallium ion beam within a cryo-FIB-SEM. The ability of gallium to form a tightly focused beam makes it well suited for this purpose. However, gallium is not suitable for higher current milling (which would enable faster lamella preparation) where the ion beam becomes more divergent (less focused) (Lucas and Grigorieff, 2023). An alternative to these conventional cryo-FIB-SEM systems are Plasma Focused Ion beam (pFIB) instruments. These instruments use ions generated from plasma generated with argon, xenon, nitrogen, or oxygen gas. Ion beams created from plasma of argon, xenon, nitrogen, or oxygen gasses have probe sizes like gallium at low voltage and are more convergent at higher voltages, making them better suited for more rapid milling of samples. Artifacts become more prominent at higher voltages however, making optimization of milling conditions an important experimental consideration. Recent work systematically details the characteristics of argon, xenon, nitrogen, and oxygen generated plasmas at different currents. Authors identify argon as the optimal gas for plasma generation due to a balance of less artifact introduction and high speed for bulk material removal (Berger et al., 2023; Dumoux et al., 2023). Both traditional cryo-FIB-SEM and pFIB techniques cause radiation damage to the exterior service of the lamella. The image deterioration due to the radiation damage can be mitigated through averaging (Lucas and Grigorieff, 2023) or using lower energies (Yang et al., 2023b).

The multiple processing steps involved in identifying regions of interest and rendering samples sufficiently thin for data collection complicates sample preparation by creating opportunities for sample contamination, devitrification, and handling damage. For this reason, it is increasingly common to integrate multiple steps of the sample preparation pipeline into a single instrument. For example, while TEM grids may be imaged in a standalone cryo-FLM and transferred to a cryo-FIB-SEM device for milling, many cryo-FIB-SEM devices now support integrated fluorescence light microscope (iFLM) to eliminate the need to transfer samples between instruments.

### 3.3 Sample preparation: High-pressure freezing (HPF)

Another widely used technique for vitrification of biological samples is high-pressure freezing (HPF), which is particularly suitable for preparing thick (>20 μm) samples. HPF rapidly vitrifies biological samples by subjecting them to high pressure and liquid nitrogen temperature in milliseconds. This rapid process minimizes the formation of ice crystals, preventing damage caused by ice crystal formation during slow freezing methods (Heiligenstein et al., 2021). As a result, HPF preserves cellular structures, organelles, and delicate molecular assemblies in a near-native frozen-hydrated state (Mcdonald, 2009). HPF effectively preserves the physiological and biochemical activities of samples due to its rapid freezing process providing a snapshot of a biochemical state of the whole cell or a group of cells and tissues.

Traditional sample preparation methods, such as resin embedding, can introduce artifacts that may impair the interpretation of experimental results. HPF preparation of the sample without any additives minimizes these artifacts by reducing structural distortions and avoiding chemical alterations (Brown et al., 2012). Additionally, modern HPF equipment can include photo- and electrical stimulation that can be applied to the sample within milliseconds before freezing (Kusick et al., 2020). Samples prepared in such a manner are suitable for a range of imaging techniques, including cryo-EM, cryo-ET, and CLEM. Unlike plunge-freezing, HPF enables comparative studies by providing a standardized and reproducible method for sample preparation. This allows researchers to compare different samples, experimental conditions, time points or genetic modifications accurately, as the preservation and freezing process is highly consistent across samples.

Two major methods downstream workflows suitable for high-resolution studies are the cryo-FIB lift-out and the Waffle method.

#### 3.3.1 Cryo-FIB lift out

Plunge freezing methods are limited to samples like single cells <10 µm of thickness to achieve complete vitrification. Cryo-Lift out combines HPF with a specialized milling workflow to address the limitation of vitrifying multicellular samples. Cryo-Lift out is an alternative method of sample milling based on mechanical manipulation rather than beam-induced material deposition aimed at *in situ* lift-out (Schaffer et al., 2019).

This method requires TEM grids that are specially engineered, a mechanical micromanipulator that is custom designed with a cryo-gripper tip. This could limit the throughput of the method. A very small percentage of the sample remains in the lamella that could lead to loss of information about the context of the lamella prepared with respect to the whole sample. A recent update on this approach addressing these limitations is Serial lift-out (Schiøtz et al., 2023). This technique creates a series of lamellae from a given volume and can capture a wholesome 3D information from a multicellular system. It is also more time efficient as compared to cryo-FIB lift out.

#### 3.3.2 Waffle method

The Waffle Method combines cryo-EM grid preparation with the advantages of high pressure freezing. For this method, a high concentration of sample is applied to grids on the grid bar side, sandwiched between two Type B HPF planchettes, and frozen using a high-pressure freezer. The thickness of the ice is dependent on the height of the grid bars, which is typically 20–25 μm, and is the limiting factor in the cell size that is used with this method. For cells larger than 25 µm or tissues, a spacer can be used to increase the overall thickness. The waffle method allows for cells to be frozen in different orientations, which contributes to the information that can be obtained from each lamella. In addition to this, the lamellae are also larger and typically include several cells; the number of cells in the lamella is dependent on cell size and concentration. Overall, the waffle method gives the user multiple advantages that leads to higher throughput in sample preparation and data collection. (Bobe et al., 2022; Kelley et al., 2022). An advantage of the waffle method over cryo-FIB lift out for the bulk samples is it does not require specialized milling equipment.

### 3.4 Complexity of instrumentation profile for cryo-ET drives the need for core centers for access and training

Cryo-ET projects can hugely vary from each other based on the target of interest and biological system. The approach for sample preparation also varies accordingly. Unlike the SPA projects where the sample and grid preparation pipeline are streamlined, for cryo-ET projects everything is dependent on the choice of system and questions being addressed. For example, early steps of viral infection can be studied with the plunge-freezing and thin cell edge imaging approach, while later stages that occur closer to the thicker, middle part of the cell, can require HPF and lamella preparation. This would require access to both plunge-freezing and HPF equipment, as well as access to a FIB instrument all for a single project. Additionally, researchers might want to use light microscopy in conjunction with tomography to take advantage of fluorescent labeling to specifically target the location of cellular events in question before TEM data acquisition. This would require access to a cryo-confocal microscope or a FIB with a fluorescent microscope integration. On the data acquisition side, the higher thickness of tomography samples as opposed to SPA requires high-end TEM instrumentation– 300 keV microscopes equipped with modern direct electron detector cameras and energy filters such as TFS Krios (Hylton and Swulius, 2021).

The requirements of pursuing sample preparation and data acquisition for a single cryo-ET project might need access to multiple sophisticated instruments, but only for a short period of time. On the other hand, data processing and analysis are crucial, which can take weeks to months per dataset. It is not feasible for every research group to invest in every instrument of the pipeline for intermittent use, while data is being processed. National centers such as NCCAT (National Center for CryoEM Access and Training) and NCITU (National Center for *In-situ* Tomographic Ultramicroscopy) provide a cost-effective solution to this problem by decoupling the sample preparation and data acquisition aspects of tomographic projects from the data processing and analysis (Zimanyi et al., 2022).

## 4 Data collection

### 4.1 Common ET considerations

Automated data collection software for SPA must be able to visit pre-programmed stage locations systematically and accurately across a grid, and at each target focus the objective lens and rapidly acquire a series of images of the specimen. Usually acquisition targets are grouped, and image shift used to acquire multiple targets per stage movement, drastically increasing throughput (Cheng et al., 2018). Data acquisition for cryo-ET is similar in principle, with the addition of sequential re-imaging of each target with varying stage tilt. In practice, however, the requirement of re-imaging an exact stage location adds several technical challenges. Microscope stages are not perfect and suffer from crosstalk between their tilt (alpha tilt) and translational shift axes (X and Y shifts), as well as backlash due to the mechanical nature of the stages. Additionally, small errors in the specimen’s eucentric height compound these issues with the result that changes in stage tilt leads to unintended stage shifts in *X*, *Y* and *Z* directions that must be corrected by a tracking routine to minimize the movement of the object of interest within the field of view of the camera. A “tracking” image is acquired after tilting the stage on a feature-rich area within the proximity to the object of interest. This image is cross-correlated with the tracking image from the previous stage tilt and the necessary adjustments to beam-image shifts are done to account for stage imperfections and to recenter the object of interest. The measured shift is corrected iteratively until the field of view is closely aligned with that of the previous tilt, then the final acquisition is done, and the stage is tilted to the next angle and the process repeats. If necessary, focus measurement and adjustment can also be done during this routine.

An additional complication in tilt series imaging is the need to spread the electron dose across many tilt images since the same area will be illuminated many times from different angles. Accumulated exposure has two relevant effects on the sample: attenuating high-resolution information by inducing small fluid motions and structure-altering chemistry, and eventually destroying the sample entirely and hence preventing tracking. The total dose must be set to maximize contrast while avoiding destruction of the sample, and then divided between tilts to maximize useful high-resolution information. Tilting of the stage increases the apparent thickness of the sample in the direction of the electron beam, decreasing signal-to-noise ratio and causing the loss of resolution (Neselu et al., 2023) thus it is desirable to acquire images from the low tilts first, where the specimen is effectively thinnest and undamaged. Historically, three tilt schedules have been used to acquire data: continuous, comprising a single sweep from across the tilt range (e.g. −60°, −57°, …, 0°, 4°, …, 60°); *bi-directional*, in which the zero-degree specimen image is collected first, followed by sequential tilts in one direction, return to zero and then sequential tilts in another direction (e.g. 0°, 3°, 6°…, 60°, 0°, −3°, −6° …, −60°); and *dose symmetric*, in which the tilt direction is alternated, usually in groups of two tilts (e.g. 0°, 3°, 6°, −3°, −6°, …, 57°, 60°, −57°, −60°) (Hagen et al., 2017). The *dose symmetric* collection scheme maximizes useful high-resolution information and is most used for projects involving STA.

### 4.2 ET software

**FIGURE 4:**
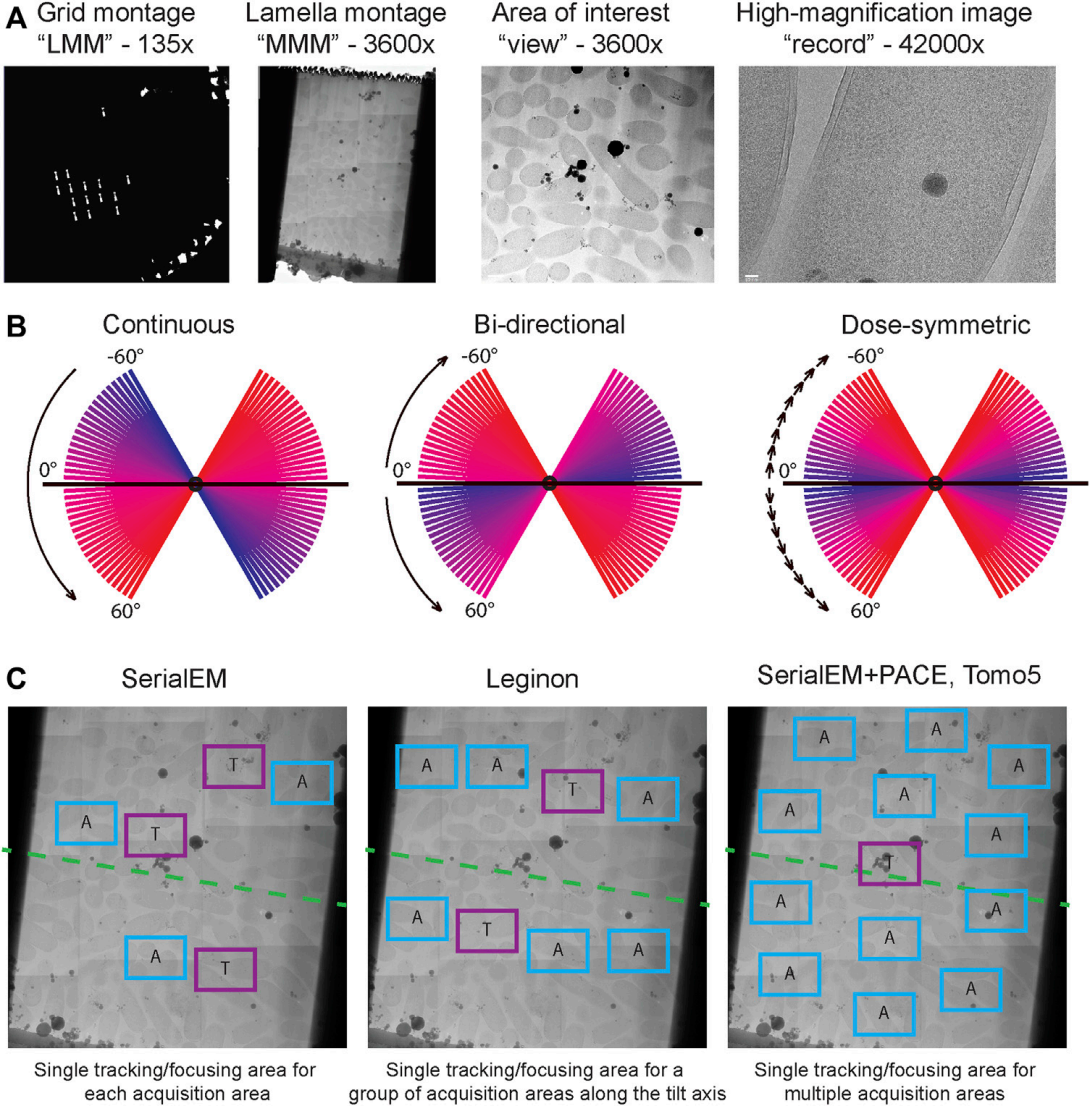
Cryo-ET data acquisition. **(A)** Typical multi-level imaging approach to tomography data acquisition. First images are acquired at the lowest magnification to obtain a grid overview or “low magnification montage (LMM); areas of interest are selected from LMM and imaged at intermediate magnification, often stitched together to produce a “medium magnification montage (MMM)”; targets are selected from MMMs and will be imaged at high magnification while tilting the stage. **(B)** Comparison of dose distribution among commonly used tilt schemes. Blue slices are earlier tilts in the sequence and have lowest total accumulated dose, red slices are latest tilts and have highest total accumulated dose. **(C)** Common strategies for object tracking during tilt series acquisition. In SerialEM each acquisition target has an associated tracking/focusing area shifted along the tilt axis. In Leginon multiple acquisition targets along a tilt axis can share a common tracking/focusing area. In PACEtomo within SerialEM a single tracking area can be shared for multiple acquisition areas without being constrained to a tilt axis; this approach is also implemented in TFS Tomo5 software.

The process of navigating to targets of interest and accurately collecting tilt series of these targets (through iterative tracking steps throughout) is historically a low throughput process. Figure 4 summarizes commonly used data collection schemes for cryo-ET. In recent years several groups applied beam-image shift approaches to collect multiple tilt series in parallel drastically increasing the throughput of tilt-series data acquisition (Bouvette et al., 2021; Peck et al., 2022; Eisenstein et al., 2023). There are many software packages that fulfill the steps required to navigate to a target of interest and collect a tilt series. Of these packages SerialEM, ThermoFisher Scientific Tomo5 and Leginon (Mastronarde, 2003; Suloway et al., 2005; Thermo Fisher Scientific, 2022) are the most popular and have their advantages and drawbacks.

SerialEM is an open-source software capable of both single-particle automated data acquisition and tilt-series acquisition. The latest release of SerialEM 4.0, has full support of Python scripting with many modules available. Conveniently, Python scripts can be incorporated into regular SerialEM scripts. Now with the support of Python scripting, SerialEM remains one of the most flexible platforms for data acquisition on TEMs. Metadata during acquisition is tracked via. mdoc files, where all microscope parameters are stored and can be retrieved when data processing using IMOD or other compatible workflows. Recently, a set of custom scripts for parallel beam-image shift based tilt series acquisition was released under the name PACEtomo (Eisenstein et al., 2023). PACEtomo significantly speeds up data acquisition by collecting images of multiple targets per single stage tilt. Amortizing the time required for stage tilt, mechanical settling, focusing, and tracking over each target group in this way typically accelerates data collection by at least an order of magnitude. Another great advantage of the PACEtomo approach is that only one tilt series per group is used for tracking via alignment of the primary target images rather than of an adjacent tracking target. Although the tracking tilt series may be less well aligned, and have greater total dose, than others within its group, the total dose on the specimen is much less as compared to traditional tilt series acquisition with unique tracking points for every target. Avoiding the accumulation of very high doses at the tracking points is helpful in avoiding large-scale damage (up to total loss) of fragile specimen areas such as FIB milled lamellae.

Leginon is another open-source software with a focus on single-particle data acquisition, but also with a tomography application built-in (Suloway et al., 2005). One of the key benefits of Leginon is the integrated web viewer, that provides on-the-fly feedback on some key parameters during the acquisition such as tracking accuracy, at-a-glance view of the images acquired so far, CTF estimation etc. Additionally, Leginon provides a convenient way to organize and track datasets and all the corresponding metadata is stored in the database and can be accessed. For data acquisition Leginon is using predictive tracking and therefore relies on the quality of the stage, especially for the early tilts, where prediction is not accurate. Leginon requires only a single focusing target with the assumption that eccentricity and focus will be unchanged along the tilt axis during the stage tilting.

Finally, Tomo5 is a commercial, closed source software designed from the ground-up for tomography data acquisition (Thermo Fisher Scientific, 2022). It has a modern GUI, it is seamlessly integrated with TFS microscopes (e.g., Titan Krios) and in the latest release it utilizes beam-image shift for high throughput tilt-series acquisition. Tomo5 is complemented by TFS Tomo Live software that provides on-the-fly feedback, pre-processing, tilt series alignment and reconstruction, and data management. As a commercial software, installation and customer support are provided but use requires a subscription and installation may require a fee. The resulting reconstructions and metadata can further be imported into downstream tomography processing workflows or can be directly used for segmentation using TFS Amira (Thermo Fisher Scientific, 2023).

## 5 Data processing

### 5.1 Computational needs for cryo-ET

Although most of the experiments and data collection can be performed at one or more national centers with little to no extra cost, one often needs to invest significantly in the computational infrastructure for data processing. Most subtomogram averaging (STA) software, such as Relion, runs on Linux operating systems, with the exception of Warp/M (Tegunov et al., 2021), which runs on Microsoft Windows. The Warp-Relion-M workflow is streamlined and powerful, therefore, a workstation dual-booted to run both Microsoft Windows and Linux is highly recommended. The Windows Subsystem for Linux (WSL) is an alternative for running Linux-based software which comes at the cost of longer processing times. Workstations require at least one modern Nvidia GPU (with >48 GB of gRAM) to run Warp and M. These two software packages will also benefit from at least 24 CPUs with 256 GB of total RAM. Storage quickly becomes an issue for cryo-ET projects, where sub-tomogram particle stacks can easily reach hundreds of gigabytes in size. Cheaper and slower hard disk drives (HDDs) are useful for long-term storage. However, processing on HDD drives can be slow due to limited read/write speeds. Read and write speeds for solid state drives (SSDs) range from 200 MB/s to 500 MB/s while for a mechanical hard drive this ranges from 80 Mb/s to 160 MB/s. Therefore, SSDs are recommended for the data processing pipeline. Additionally, having multiple GPUs on the workstation is beneficial, as STA in Relion can be time-consuming. One way to minimize the need for extra investments is to utilize the available computational resources for single particle analysis, such as GPU clusters with Relion installed. Another option is to utilize cloud computing resources like Amazon Web Services (AWS) or Cryo-EM Open Source Multiplatform Infrastructure for Cloud Computing (COSMIC2) (Cianfrocco et al., 2017), although throughput and control of the processing environment can be limited.

In this section, we aim to provide a simplified overview of a data processing workflow in cryo-electron tomography (cryo-ET) that will hopefully serve as a starter guide.

The steps in single particle analysis (SPA) and cryo-ET data processing are similar during pose refinement and classification. Additionally, there are three steps specific to tomography: tilt series alignment, tomogram reconstruction, and 3D particle picking. For beginners, it is helpful to use software packages that implement a complete cryo-ET workflow, such as EMAN2 (Chen et al., 2019), and Dynamo (Castaño-Díez et al., 2012), which come with a user-friendly graphical user interface. Other software packages such as emClarity (Himes and Zhang, 2018), TomoBear (Balyschew et al., 2023), Relion-4 tomo (Zivanov et al., 2022), Warp-Relion-M (Tegunov et al., 2021), and TomoMan/STOPGAP (https://github.com/williamnwan/TOMOMAN/, https://github.com/williamnwan/STOPGAP) rely on IMOD (Mastronarde and Held, 2017) or AreTomo (Zheng et al., 2022) for tilt series alignment. 3D particle picking is still a challenging step that usually needs investigating in multiple software packages for the best outcome per dataset. Additionally, these packages (except for Dynamo) implement sub-tilt refinement, an important step in high-resolution STA in their own way.

**FIGURE 5:**
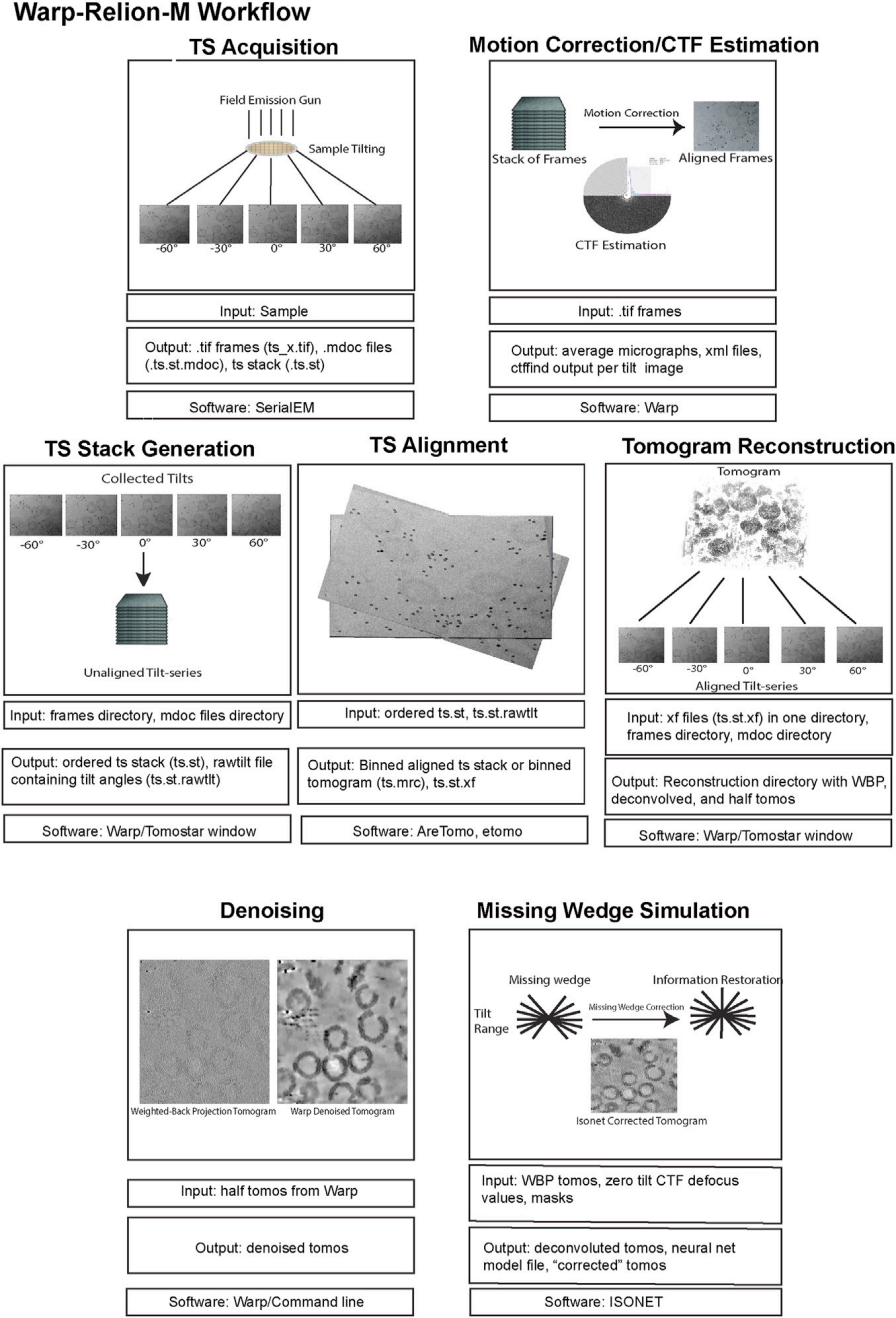
The data pre-processing workflow of cryo-ET with STA. Data pre-processing workflow using the publicly available dataset EMPIAR-10164 as an example. Representative inputs and outputs of each step are shown as an image in the top panel. Input and output file formats are listed below, followed by the software used in the step from motion correction of frames to tomogram generation.

**FIGURE 6:**
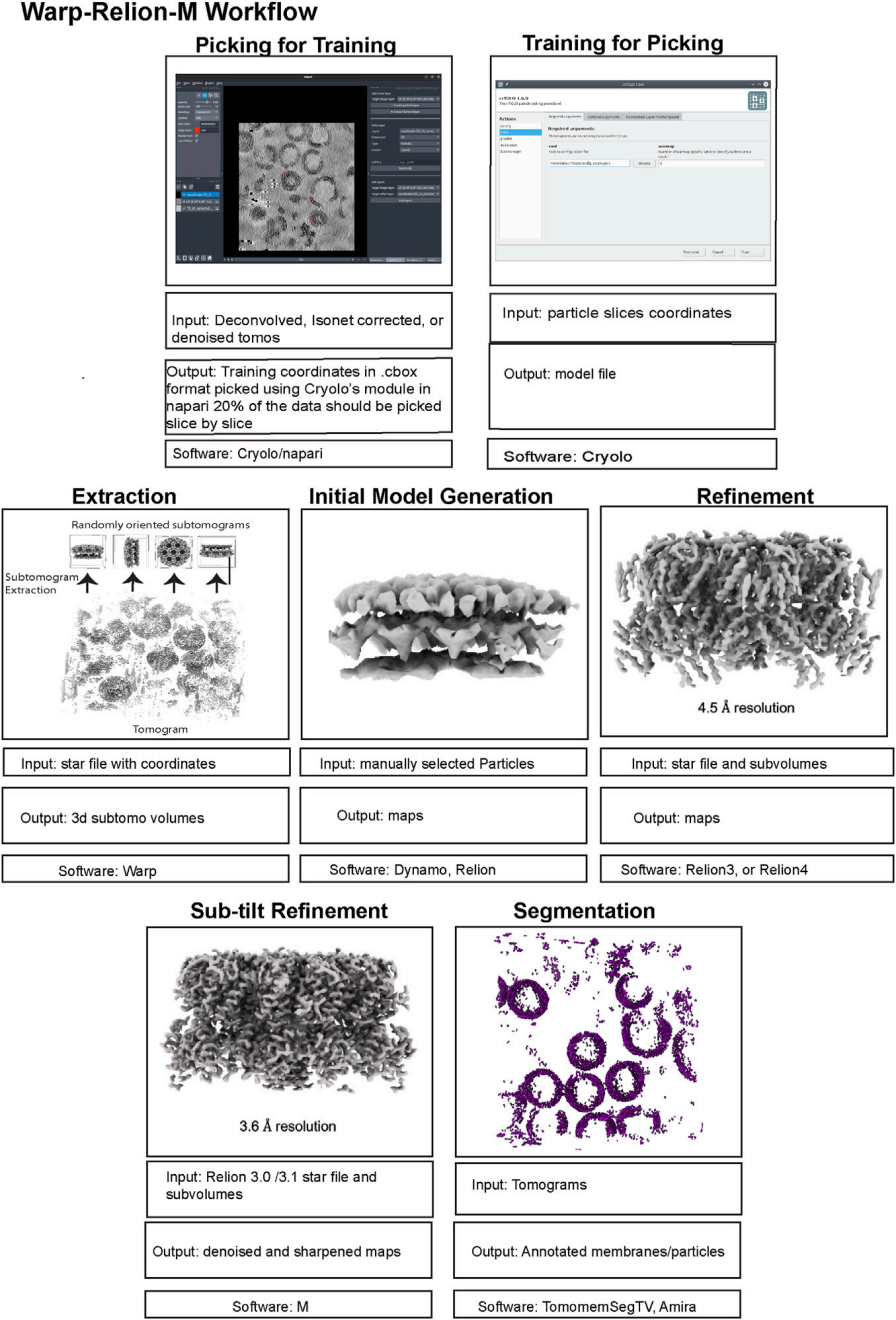
The data post-processing workflow of cryo-ET with STA. A continuation of Figure 5 for the post-processing workflow of cryo-ET with STA, with the publicly available dataset EMPIAR-10164. Representative inputs and outputs of each step are shown as an image in the top panel. Input and output file formats are listed below, followed by the software used in the step from particle picking to sub-tilt refinement.

Here, we review a more commonly used workflow that includes: AreTomo, Dynamo, Warp/M, Relion. Recently, the Warp-Relion-M workflow has gained significant attention due to its relatively user-friendly workflow and ability to achieve high-resolution structures. Therefore, we will primarily concentrate on this approach to introduce various aspects of cryo-ET data processing (Figures 5, 6). For more detailed documentation of the method, readers can refer to teamtomo.org.

### 5.2 Initial processing - from frames to particle picking

#### 5.2.1 Preprocessing

The preprocessing stage, like SPA, involves motion correction and CTF estimation of raw movie frames and can be calculated through a variety of methods. The Warp software offers convenient on-the-fly preprocessing (Tegunov and Cramer, 2019), which currently runs on Microsoft Windows, therefore, requiring a dedicated machine. During this step, Warp generates motion-corrected and averaged micrographs for each tilt and assembles each tilt series into a stack with an additional text file containing the nominal tilt angles. It is important to note that for this assembly Warp requires data collection metadata in the mdoc format, specific to data collected by SerialEM (Mastronarde, 2005) and the Thermo Fisher Scientific (TFS) software TOMO5 (Thermo Fisher Scientific, 2022) https://www.thermofisher.com/us/en/home/electron-microscopy/products/software-em-3d-vis/tomography-software.html, although users can manually generate mdoc files if necessary. Alternatively, preprocessing can be done outside of WARP using MotionCor2 (Zheng et al., 2017). Warp’s GUI allows for the visualization of data statistics like defocus, resolution estimates, and drift which is helpful in catching data collection issues. The output of preprocessing, regardless of which software is used, must yield a stack file containing all the motion and CTF corrected images which in combination with an mdoc or rawtlt file can be used for tilt series alignment.

#### 5.2.2 Tilt series alignment and tomogram reconstruction

Tilt series alignment compensates for stage movement errors during tilting. Warp cannot do tilt series alignment therefore, the preprocessed stack file must be moved into another software for alignment, then back into Warp before processing can continue.

AreTomo (Zheng et al., 2022), is a robust and easy-to-execute fiducial-free alignment tool that uses GPU processing and aligns a tilt series quickly. Batch processing with AreTomo requires some bash or python scripting but can be useful when dealing with large data sets. At this step, the most important parameter to fine-tune is the alignment thickness (-AlignZ parameter) which is a value that should approximate the actual sample thickness. Therefore, a few rounds of alignment using different thickness values is required, keeping in mind that this can vary for different tilt series within the same session. AreTomo also has an ROI (region of interest) function allowing a user to choose an object to be preferentially aligned within the tilt series. Alternatively, Dynamo (Castaño-Díez et al., 2012) or IMOD (Mastronarde and Held, 2017) can be utilized for tilt series alignment through fiducials. The important metadata files created from AreTomo or IMOD alignments are the refined tilt angles in the tlt file and the shifts and rotations in the xf file. These can be used in other software to generate an aligned stack and the final reconstructed tomogram.

While all these packages can also generate back-projected tomograms after tilt series alignment, only the aligned tilt series metadata in the form of xf files are imported back into Warp for enhanced geometric CTF estimation and weighted back-projection (WBP) tomogram reconstruction (Penczek, 2010). A common problem in Warp tomogram reconstruction is that the smallest alignment errors that are hard to diagnose by eye can translate into very poor reconstructions. This can happen even when other reconstruction algorithms like AreTomo, IMOD, or Tomo3d′s WBP, SART, or SIRT produce a good tomogram for the same tilt series alignment. This problem is generally solved by changing the alignment thickness parameter and turning off the sample tilt correction (-TiltCor parameter) in AreTomo. The improvement must be inspected manually for AreTomo. On the other hand, IMOD provides alignment scores that could be used as a guiding reference.

WBP tomograms must be used for downstream STA and therefore, they lack proper filters that enhance the low-resolution spatial frequencies which are commonly desired for particle picking and segmentation. To aid with these processes, Warp generates CTF-deconvolved tomograms, as well as even and odd (tilt) half-reconstructions. Both of these tomograms can be used for better visualization of objects within the tomogram. Warp has an excellent denoising algorithm (noise2noise) that can be run from Windows PowerShell to increase the contrast of your sample within a tomogram (Lehtinen et al., 2018). Although unbinned/lower binning is required for high resolution structure determination, it is important to mention that highly binned tomograms with a pixel size of 10–20 Å (bin by 8) are adequate for general visualization, segmentation, and particle picking. Smaller pixel sizes will severely reduce the speed while improving the outcome only marginally if at all.

#### 5.2.3 Particle picking and extraction

Probably the most important advantage of tomography over SPA is the ability to pick particles in 3D and therefore, avoiding all the tracing and picking difficulties that arise from overlapping particles in a single projection, especially in a cellular context. Software like Dynamo provide a plethora of tools for such 3D particle picking by taking advantage of the geometric properties of the sample. However, this process can be time-consuming for larger datasets or if your protein of interest lacks a consistent geometry. Machine learning approaches like crYOLO (Wagner et al., 2019), TomoTwin (Rice et al., 2023), and EMAN2 (Chen et al., 2017) have sped up this process. For crYOLO and EMAN2, the training dataset must be manually picked. For crYOLO, the size of the training dataset usually scales with the size of the full dataset. TomoTwin, on the other hand, is pre-trained and is designed to readily find most of the particles in a heterogeneous dataset.

Once the coordinates of the particles are determined, the particles can be extracted in three major different ways. Conventionally, the particles can be direct 3D extractions from the tomograms. A classical particle alignment and refinement of such sub-volumes in Relion can result in high enough resolution for the visualization of bulky side chain densities (Bharat and Scheres, 2016), given proper CTF corrections (Turoňová et al., 2017). However, achieving high resolution with this approach is limited to a few cases. To overcome this resolution barrier, software such as M (Tegunov et al., 2021), EMClarity (Himes and Zhang, 2018), and EMAN2 (Chen et al., 2019) deploy the so-called sub-tilt refinement which generates 2D tilt stacks for each particle to further refine and correct aberrations and deformations at each 2D tilt image. This also alleviates the errors introduced by the interpolations in each 3D subtomogram back-projection. This brings us to a second approach to particle extraction which uses 2D tilt-stacks of particles from the beginning, as implemented in TomoBear and Susan (Balyschew et al., 2023). One advantage of this approach is efficient memory usage which in turn allows for processing larger particles and box sizes without using GPUs. The third approach for particle extraction is implemented in Relion 4 Tomo (Zivanov et al., 2022). The extracted sub-volumes are called pseudo-subtomograms. As opposed to regular subtomograms that are real space back-projections of the particles, pseudo-subtomograms are made of Fourier transforms of the 2D images inserted into a 3D Fourier space based on the initial tilt information. This was adapted so that existing Relion refinement algorithms could be used, resulting in a workflow and a platform that is very similar to SPA.

### 5.3 Subtomogram averaging workflow–from particle stacks to reconstructions

#### 5.3.1 Initial model generation

The first step in STA begins with initial model generation, as a template is required for initial alignment of the subtomograms that were extracted from the tomogram. The initial model or reference can be a previous structure that was determined from another method such as X-ray crystallography or single-particle cryo-EM which have been low pass filtered. Though, oftentimes a present structure might not be available that can be readily used as an initial model for STA, and thus, it is necessary to generate a *de novo* initial model. There are several different software packages that can be used to generate an initial model for STA such as EMAN2, Relion, and Dynamo. The process typically begins with the user manually picking a small subset of ∼50–500 particles from the tomograms, followed by extraction of the particles and generation of a *de novo* initial model from the manually picked set of particles. Similarly, to single particle analysis, the software packages EMAN2 and Relion use stochastic gradient descent (SGD) to generate initial models, with the ability to output multiple classes which can be useful as an additional cleaning step to remove poor particles in heterogeneous datasets. Dynamo follows a template-free procedure, where the subset of manually picked, particles are subjected to a “random model” protocol. This involves creating multiple, different volumes (often spheres or cylinders) which are used as initial models for alignment against the particle subset. The process uses the principle of cross-correlation for alignment and involves iteratively aligning each of the random volumes to each of the particles in the subset. At the end of each iteration, an average volume is calculated from the highest scoring alignments. This average volume is then used as a reference model for the next iteration. The process is repeated until the changes between iterations become minimal, indicating that the model has converged to a stable solution. The quality and accuracy of the initial model is critical STA as it directly impacts the precision of the subsequent alignments, the ability to identify and classify different conformations in heterogeneous samples, and ultimately determines the resolution and reliability of the final 3D reconstruction.

#### 5.3.2 Refinement

Once the initial model has been generated successfully, the next step in the workflow is refinement. In this phase, the initial model serves as a reference to align the extracted subtomograms from the tomogram. In Relion, refinement for cryo-electron tomography is more intricate than single particle analysis. Instead of aligning 2D particles to 2D projections, 3D subtomograms are aligned to a 3D reference, involving both rotations and translations. This alignment is driven by Relion’s Bayesian approach, utilizing regularized likelihood optimization. If the structure under investigation possesses symmetry, this can be employed during refinement to enhance the signal-to-noise ratio. A unique challenge in cryo-ET is the “missing wedge” phenomenon due to the restricted tilt range in tomographic data collection. Relion incorporates strategies to address these challenges during refinement. After achieving convergence through iterative alignment and averaging, Relion outputs a star file detailing the refined poses for the subtomogram alignments. For further refinement, especially sub-tilt refinement aiming for higher resolutions, complementary software tools, such as package “M”, can be employed.

#### 5.3.3 Sub-tilt refinement

The M software suite includes a component specifically designed to refine the alignment of the tilt-series, termed sub-tilt refinement. In contrast to traditional approaches which align the tilt-series as a whole, sub-tilt refinement aligns smaller subregions of the tilt-series independently. This allows for correction of local distortions and inaccuracies in the tilt geometry, improving the overall alignment of the tilt-series and leading to more accurate and higher resolution reconstructions. This is particularly important for datasets with a high-tilt range, or those that are suffering from specimen deformation of beam-induced motion. By using a local refinement approach, M can correct for these issues and produce a more reliable, accurate reconstruction of the map. M takes a Relion3.0/3.1 formatted starfile containing the previously refined poses, the corresponding half-maps, and a binary mask. M can greatly improve the resolution of the reconstructions obtained from the refinement step, though can oftentimes get stuck in a local minimum, and thus requires running different parameters in a stepwise fashion.

### 5.4 Segmentation and visualization

Segmentation can provide information about the membrane morphologies and the localization and organization of large macromolecules (>0.5 MDa) to the extent that the Euler angles are not required for the analysis. While particle picking and extraction are commonly conducted on unfiltered, WBP tomograms, segmentation can utilize any kind of reconstructed tomogram. For better visualization, the missing wedge can also be “simulated” computationally by EMAN2, Isonet (Liu et al., 2022), REST (Zhang et al., 2023a), or Warp’s Noise2Tomo leading to increased contrast and signal over noise. However, users should take caution in trusting and interpreting their observations since these packages fill in the missing wedge with data that is computationally derived and not “real”. The subsequent segmentation of densities within tomograms can be done in different software packages depending on the types of structures one hopes to extract. Dragonfly (Heebner et al., 2022) and DeePict (De Teresa-Trueba et al., 2023) are neural networks designed to segment both macromolecules and membranes. For membrane-only semi-automated segmentation, one can use TomoSegMemTV (Martinez-Sanchez et al., 2014). This package is designed to enhance the membrane signal and segment the membranes using tensor voting. However, in the case of tomograms that contain several membranes, TomosegmemTV sometimes assigns the same object number to neighboring membranes, mostly because of tomography artifacts. This can be manually fixed in Amira or Avizo which can be followed by more specific labeling within each object or membrane within the tomogram. With such labeling and segmentation at hand, one can then use surface morphometrics analyses to quantify membrane characteristics such as curvature, distances, etc. which can be very powerful when it is used to provide context for STA.

### 5.5 Data processing Summary

Cryo-ET data processing is a complex, multi-step endeavor that presents several challenges and opportunities. This guide provides an overview of the steps involved, from preprocessing through refinement, and the various software tools available to facilitate these processes. The Warp-Relion-M workflow stands out for its user-friendliness and the high-resolution structures that it can achieve (Figures 5, 6). This guide also highlights the importance of making informed decisions throughout the data processing pipeline, such as choosing between different particle extraction methods, understanding the limitations and strengths of segmentation tools, and adapting strategies for initial model generation and refinement. While the insights provided here should serve as a useful foundation for novices to navigate the vast landscape of cryo-ET data processing, it is also important to note that cryo-ET data processing is very much under active development, considering it is a major bottleneck in the cryo-ET workflow. Additionally, data processing is highly sample dependent and one validated workflow may not work at all between two seemingly similar samples making it one of the most challenging aspects modern cryo-ET faces. Much work needs to be done to reduce the storage requirements necessary for processing large datasets, increasing the speed at which STA can be performed, better streamlined processing methods that reduce the necessity of the user to have to script between differing software packages and better particle picking algorithms that reduce the necessity for users to have to spend weeks to months manually picking macromolecules of interest.

## 6 Data deposition and archiving

The Coulomb potential maps generated during cryo-EM and cryo-ET workflows can be deposited to the Electron Microscopy Databank (EMDB) (https://emdb-empiar.org) (Lawson et al., 2016), whilst the raw data from which the maps or tomograms are derived can be archived in the Electron Microscopy Public Image Archive (EMPIAR) (https://empiar.org) (Iudin et al., 2023). When a coordinate model is built into an EM map it can be deposited to the Protein Data Bank (PDB) (https://www.wwpdb.org/) (wwPDB consortium et al., 2019). These archives promote the free and enduring access to structural biology data for all.

**FIGURE 7:**
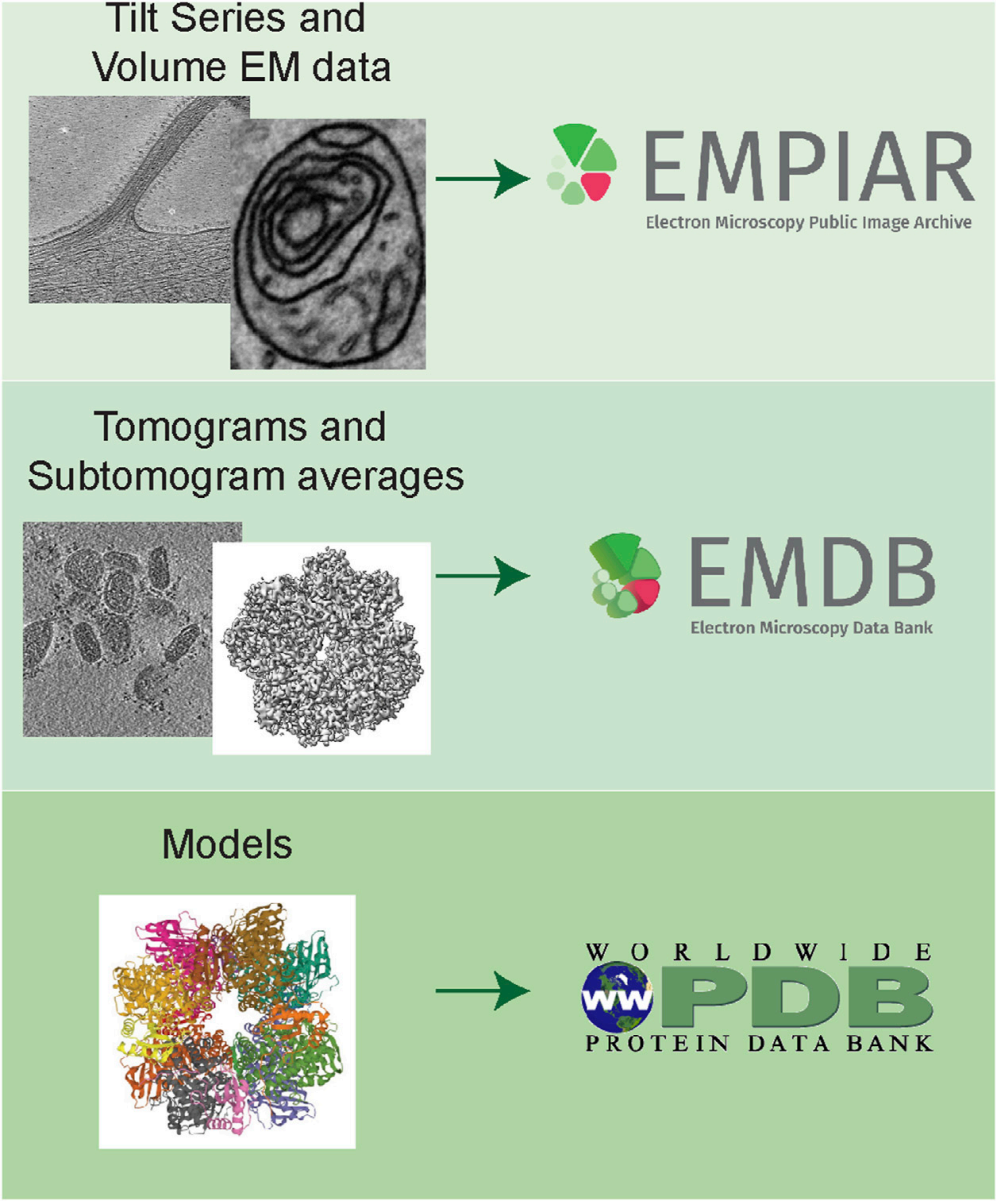
Archiving of volumeEM, cryo-ET and STA data. Tilt series and volumeEM data are archived in EMPIAR, EMPIAR-11456 (unpublished) and EMPIAR-11449 (Suga et al., 2021) shown in this section. Tomograms and subtomogram averages are archived in the EMDB, EMDB-15182 (Calder et al., 2022) and EMD-14590 (Ni et al., 2022) shown in this section. Finally, models are deposited to the PDB 7ZBT shown in this section (model associated with EMD-14590).

In addition to cryo-EM and cryo-ET data, EMPIAR archives data from several other methodologies including volume electron microscopy (volumeEM) and soft x-ray tomography, the full list can be found in Section 1.2 of the EMPIAR policy document (https://www.ebi.ac.uk/empiar/policies). The recommended deposition site for each data type is described in Figure 7.

## 7 Discussion

A combination of the major recent advances has made it possible to achieve sub nanometer resolution in structures from a crowded cellular environment or other complex systems.

Sample preparation methods for cryo-ET have revolutionized the field in the last few years. Micropatterning is a useful tool to target cells directly on the grids at a precise location (Sibert et al., 2021a; Sibert et al., 2021b). Sample thinning methods like serial lift-out and waffle method utilize HPF demonstrate the potential of handling multicellular and tissue samples (Bobe et al., 2022; Schiøtz et al., 2023). Lamellae preparation could be challenging without a complementary tool like light microscopy. Yang et al. (2023a), developed a workflow for 3D correlative cryo-FLM-FIB-ET that precisely targets to ROIs with iFLM and cryo-FIB SEM. Pairing of super-resolution light microscopy and correlated electron microscopy is further being strengthened by optimal support grids (Last et al., 2023). Recently, Xenon plasma FIB milling was used to make lamella directly from human brain tissue derived through biopsy to visualize sub-cellular features (Creekmore et al., 2023). Additionally, with the development of plasma-FIBs, volumeEM is gaining traction. In this approach a thin slice of the sample is ablated and the fresh surface is scanned with SEM producing as high as ∼20 nm resolution images (Dumoux et al., 2023). By continuously shaving off a top layer and scanning the layer beneath, volumeEM allows visualization of large volumes such as whole cells, tissues and small organisms at intermediate resolution bridging the gap between light microscopy and cryo-ET (Kelley et al., 2023). VolumeEM and cryo-ET approaches can be combined, where the sample is first thinned while continuously imaged with SEM to identify the ROI producing a thin lamella suitable for cryo-ET (Dumoux et al., 2023).

Machine Learning (ML) and Deep Learning (DL) is being used to increase throughput and improve data analysis in cryo-ET. Template matching is making it possible to identify and extract out particles irrespective of high background (Zhang et al., 2023b). pyTOM package also considers extensive angular sampling in template matching (Chaillet et al., 2023). Flexibility and heterogeneity can be sampled using the latest packages-tomoDRGN (Powell and Davis, 2023), cryoDRGN-ET (Rangan et al., 2023).

In recent years, cryo-EM has played a pivotal role in advancing structural virology, with their significance becoming even more apparent in the context of emerging infectious diseases. Cryo-ET is rapidly maturing as a technology, catalyzing significant advancements in our understanding of viral infection biology. Several groups have recently reviewed cryo-ET advances in studying viral infections (Graham and Zhang, 2023; Hong et al., 2023; Zimmermann and Chlanda, 2023)**.**

As we look into the future of cryo-ET, the emphasis lies in enhancing throughput through the integration of a variety of biophysical fields from advanced light microscopy tools to data analysis strategies. Cryo-ET is constantly pushing the resolution limits *in situ* (O’Reilly et al., 2020; Gemmer et al., 2023). These innovations are poised to expedite the pace of discovery, offering profound trends that cryo-ET will continue to be at the forefront of structural infectious disease research, pointing the way toward innovative strategies for diagnosis, treatment, and prevention.
